# Comparative analysis of metabolic characteristics and prognostic stratification of HER2-low and HER2-zero breast cancer using ^18^F-FDG PET/CT imaging

**DOI:** 10.1186/s40644-024-00812-6

**Published:** 2024-12-18

**Authors:** Yuan Gao, Lei Yin, Linlin Ma, Caixia Wu, Xiaojuan Zhu, Hongjin Liu, Li Liang, Jinzhi Chen, Yulong Chen, Jingming Ye, Ling Xu, Meng Liu

**Affiliations:** 1 Department of Nuclear Medicine, Peking University First Hospital, No.8, Xishiku Street, West District, Beijing, 100034 China; 2 Thyroid and Breast Surgery, Peking University First Hospital, Beijing, China; 3 Department of Pathology, Peking University First Hospital, Beijing, China

**Keywords:** ^18^F-FDG PET/CT, Breast cancer, HER2-low, Prognosis, Antibody-drug conjugates (ADCs)

## Abstract

**Background:**

Recent advancements in novel anti-human epidermal growth factor receptor 2 (HER2) antibody-drug conjugates (ADCs) have highlighted the emerging HER2-low breast cancer subtype with promising therapeutic efficacy. This study aimed to comparatively analyze the metabolic characteristics and prognostic stratification of HER2-low and HER2-zero breast cancer using baseline fluorine-18 fluorodeoxyglucose (^18^F-FDG) positron emission tomography/computed tomography (PET/CT) imaging.

**Methods:**

Consecutive patients with newly diagnosed breast cancer who underwent ^18^F-FDG PET/CT prior to therapy in our hospital were retrospectively reviewed. The relationship between metabolic parameters (maximum standardized uptake value (SUVmax), tumor-to-liver SUV ratio (TLR), total lesion glycolysis (TLG), and metabolic tumor volume (MTV)) in primary lesions and HER2 expression was analyzed. The survival analyses were performed to identify the prognostic factors for disease-free survival (DFS) in patients with HER2-negative (HER2-low versus -zero).

**Results:**

In total, 258 patients (mean age: 54 ± 12 years) were included. In hormone receptor (HR)-positive subgroup, SUVmax and TLR were significantly higher in HER2-low than in HER2-zero (*P* = 0.045 and 0.03, respectively). But in HR-negative subgroup, there was no significant metabolic difference between HER2-low and HER2-zero (All *P* > 0.05). The four metabolic parameters were significant predictors of DFS in HER2-negative patients (All *P* < 0.01), but there was no significant difference in DFS between HER2-low and -zero, regardless of tumor metabolism. Moreover, in HER2-zero patients, the DFS of patients with high metabolism was significantly shorter than that of patients with low metabolism (*P*_SUVmax_ = 0.002, *P*_MTV_ = 0.03, *P*_TLG_*=* 0.005, *P*_TLR_ < 0.001, respectively), but without a similar finding in HER2-low patients.

**Conclusion:**

Our study demonstrated the HR-positive HER2-low breast cancer exhibited a particularity in glucose metabolic profile. Additionally, HER2-zero patients with elevated metabolism were associated with inferior prognosis and warranted careful attention in clinical evaluations.

## Background

Given the encouraging results achieved by human epidermal growth factor receptor 2 (HER2)-targeted antibody-drug conjugates (ADCs) in breast cancer patients with HER2-low [1], it is important to fully understand the biological and clinical features of this subgroup [2, 3], as well as noninvasively stratify patients prior to treatment. So far, some studies have attempted to explore the differences in clinicopathological characteristics, biological behaviors, and prognosis between patients with HER2-low and -zero, but no consistent conclusions have been drawn to support HER2-low as an independent subtype [4–6].

The introduction of HER2-low changes the categorization of HER2 expression profiles, transforming from a dichotomous classification of HER2-negative versus -positive to a trichotomous classification of HER2-zero, -low, and -positive. Recently, multiparametric MRI radiomics has been performed to distinguish HER2-low from HER2-negative breast cancers [7, 8]. However, the characteristics regarding the glucose metabolic profiles in breast cancer with HER2-low, particularly compared to HER2-zero, has not been explored.

As a noninvasive technique to detect glucose metabolism in lesions, ^18^F-FDG PET/CT is increasingly being applied in the systemic evaluation of breast cancer, especially in pretreatment staging and therapeutic monitoring of advanced breast cancer [9]. In the process of tumor progression, proliferative tumor cells undergo metabolic remodeling and depend on glucose metabolism heavily, resulting in increased FDG accumulation in primary or metastasis lesions [10]. Since the expression of HER2 is involved in regulating the proliferation, survival, and metastasis of tumor cells [11], it is speculated that various HER2-expressing populations may exhibit different glucose metabolic phenotypes.

Significantly, HER2 expression exhibits temporal and spatial heterogeneity, specifically reflected in HER2 expression changes throughout treatments (temporal heterogeneity), differences at various locations within the same tumoral lesion (spatial intralesional heterogeneity), and inconsistencies between primary and recurrent/metastatic lesions (spatial interlesional heterogeneity), which might impact treatment response and resistance [12, 13]. ^18^F-FDG PET/CT, as a non-invasive, dynamic, and whole-body examination, has potential advantages in reflecting tumor heterogeneity.

To date, some studies have shown that breast cancer with HER2 overexpression is characterized by high glucose metabolism [14–16]. Other studies have indicated that elevated baseline metabolism in primary tumors is associated with poor prognosis in patients with hormone receptor (HR)-positive HER2-negative breast cancer [17, 18]. To the best of our knowledge, the potential prognostic value of baseline ^18^F-FDG PET/CT in HER2-low patients is still unclear.

In this study, we tried to comparatively analyze the metabolic characteristics of HER2-low and HER2-zero breast cancer using baseline ^18^F-FDG PET/CT. Besides, the prognostic stratification of metabolic parameters in patients with HER2-low and -zero were analyzed, in order to explore the potential value of ^18^F-FDG PET/CT in the hierarchical management of these patients.

## Materials and methods

### Patient characteristics

The data of consecutive breast cancer patients, who underwent ^18^F-FDG PET/CT examination for evaluating of possible metastasis from January 2017 to October 2021 in our hospital, were retrospectively reviewed.

The inclusion criteria were as follows: (1) newly diagnosed breast cancer by primary tumor biopsy or surgical pathology; (2) a baseline ^18^F-FDG PET/CT examination performed within two months prior to treatment. The exclusion criteria were as follows: (1) the data of immunohistochemistry (IHC) or fluorescence in situ hybridization (FISH) of HER2 missing; (2) Carcinoma in situ.

For further prognostic analysis, HER2-low and -zero patients in stage I-III who received operations were included. To remove confounding factors, the additional exclusion criteria were proposed as follows: (1) lost to follow-up; (2) presence of other synchronous primary malignancy or a history of malignancies; (3) bilateral synchronous breast cancer.

The documented clinicopathological parameters included age, gender, body mass index (BMI), menopausal status, tumor node metastasis stage [19], tumor histology, histologic grade, estrogen receptor (ER), progesterone receptor (PR), Ki-67, molecular classification, and therapeutic regimen. The metastatic lymph nodes were determined by baseline pathological results, including core needle biopsies and surgical specimens.

Patients’ records were anonymized and de-identified before analysis. The retrospective data collection and analysis procedures were approved by the ethics committee of our hospital, waiving the need for written informed consent.

### Imaging analysis of 18F‑FDG PET/CT

As described in previous studies [20], all patients fasted for at least 6 h before ^18^F-FDG PET/CT examination, and the images were acquired and independently reviewed by two experienced senior nuclear medicine physicians, who were blinded to all patients’ information. If the results differed, they discussed the findings and then reached a consensus.

According to the ^18^F-FDG PET/CT images, the metabolic parameters of primary tumor, including maximum standardized uptake value (SUVmax), mean standardized uptake value (SUVmean), and metabolic tumor volume (MTV), were measured by outlining a volume of interest, which was carefully put on the primary lesion to encompass the entire tumor. The parameters of SUVmean and MTV were calculated using an SUV threshold of 40% of SUVmax [21]. SUVmean of the liver was measured by drawing a volume of interest in the center of an area of non-diseased right hepatic lobe (diameter of 3 cm) [22]. Total lesion glycolysis (TLG) was automatically obtained as MTV multiplied by SUVmean, and tumor-to-liver SUV ratio (TLR) was defined as tumor SUVmax divided by liver SUVmean [23].

In the case of unilateral multifocal tumors, the parameters of the highest metabolic tumor were used for prognostic analysis.

### Immunohistochemical evaluation

The results of hematoxylin and eosin staining (HE) and IHC were independently reviewed by two pathologists who were unaware of the outcomes. Differences in diagnosis between the two pathologists were resolved by re-reviewing the biopsies to reach a consensus. ER and PR expression were classified as positive when clear cell membranous staining ≥ 1% [24]. HR-positive means ER-positive, PR-positive, or both. HER2 expression was judged according to the HER2 detection standard [25]. IHC 0 was considered as HER2-zero, IHC 1 + and IHC 2 + with FISH (-) were HER2-low, while IHC 2 + with FISH (+) and IHC 3 + were HER2-positive. HER2-negative included both HER2-low and HER2-zero. Ki-67 ≥ 30% was considered the threshold for the classification of high and low Ki-67. The histologic grades were evaluated based on the Nottingham grading system [26]. Based on the expression of ER, PR, HER2, and Ki-67, breast cancer was divided into four molecular types [27].

### Follow up and clinical endpoints

Follow-up surveillance after operation included breast and axillary lymph node ultrasound, breast MRI, abdominal ultrasound, abdominopelvic CT, chest X-ray, chest CT, and laboratory data. During the follow-up period, the information of every patient was first collected from 3 months to 6 months after operation, then regularly collected every 6 months for the first 3 years, and annually afterward. Gynecologic ultrasound was reviewed annually in patients receiving endocrine therapy.

Disease-free survival (DFS) was defined as the time from the date of primary surgery to the date of the radiological or histological evidence of recurrence for the first time, death of any cause, or censored at the last follow-up, whichever came first [6].

### Statistical analysis

Continuous data were expressed as medians with interquartile range (IQR) in parentheses or mean ± standard deviation (SD), and categorical variables were shown as numbers (percentages). Analysis of variance (ANOVA), Kruskal-Wallis test, Chi-Squared test or Mann-Whitney U test was carried out to compare the variables between different groups, respectively. Bonferroni correction was applied in multiple comparisons. Jonckheere-Terpstra test (J-T test) was used for trend testing.

Receiver operating characteristic (ROC) curves were analyzed for the optimal cutoff value and area under the curve (AUC) for the continuous variables. Survival analysis was evaluated by Kaplan-Meier analysis, and the comparison of different Kaplan-Meier curves was performed using log-rank test and Bonferroni correction. Cox proportional hazard analyses were undertaken to identify the prognostic factors for DFS, and the hazard ratios (HR) and 95% confidence intervals (CI) of the predictors were acquired.

All statistical analyses were executed using the SPSS 26.0 software (SPSS Software Inc., Chicago, IL, USA) and R version 4.2.3 software (R Core Team 2023. R Foundation for Statistical Computing, Vienna, Austria.). *P* < 0.05 was considered statistically significant.

## Results

### General characteristics

**Fig. 1:**
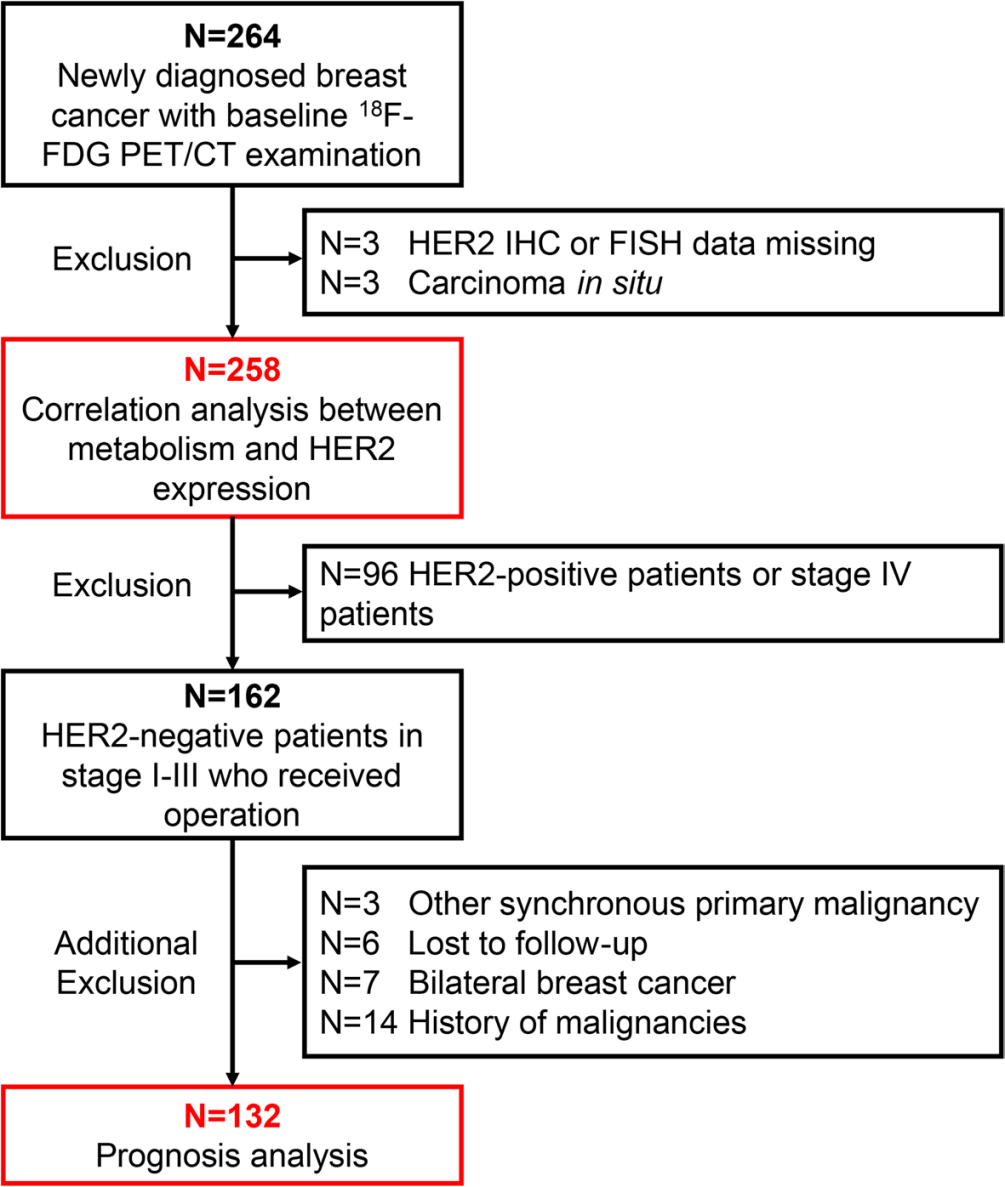
Flowchart of patient inclusion and exclusion

A total of 258 patients with newly diagnosed breast cancer were included (Fig. 1), including 256 women (99.2%) and 2 men (0.8%), with a mean age of 54 ± 12 years (range: 26–90 years) (Table 1). Of the 258 cases, 245 cases had a single lesion, 6 cases had unilateral double lesions, and 7 cases had bilateral lesions. Thus, the histopathological results were obtained from a total of 271 primary tumors.

**Table 1:** Clinical characteristics of total patients

Characteristic	Distribution
**Age**,** years (± SD)**	54 ± 12
**Gender**	
Female	256 (99.2)
Male	2 (0.8)
**BMI**	24.1 (22.0–26.4)
**Menopausal status**^a^	
Postmenopausal	152 (59.4)
Premenopausal	101 (39.5)
Unknown	3 (1.1)
**Relatives with BC**	
Present	24 (9.3)
Absent	234 (90.7)
**Tumor focality**	
Single focal BC	245 (95.0)
Unilateral double focal BC	6 (2.3)
Bilateral BC	7 (2.7)
**Stage at BC diagnosis**	
stage I-III	231(89.5)
Stage IV	27 (10.5)
**Treatment for stage I-III BC**	
Adjuvant	188 (72.9)
Neoadjuvant	135 (52.3)
Operation	231 (89.5)

Qualitative data are numbers followed by percentages in parentheses; continuous data are median followed by interquartile range (IQR) in parentheses, except for age

Abbreviations: *BMI* body mass index, *BC* breast cancer

^a^Two men were excluded

One hundred and thirty-two female patients with HER2-low and -zero in stage I-III who received operation were selected for prognostic analysis (Fig. 1), with a mean age of 54 ± 12 years (range 26–85 years). The median follow-up time was 23 months (range: 4–57 months). Sixteen patients experienced recurrence or death, totally accounting for 12.1%. The main recurrence sites are bone (7 sites), liver (6 sites), lung (5 sites), and regional lymph nodes (4 sites).

### Correlations of metabolic parameters and clinicopathological characteristics with HER2 expression

A total of 271 primary tumors were analyzed, including 62 lesions in HER2-zero (22.9%), 120 lesions in HER2-low (44.3%), and 89 lesions in HER2-positive (32.8%) group, respectively. As shown in Table 2, the parameters of primary tumor SUVmax, TLR, histologic grades, ER, PR, Ki-67 and distant metastasis showed significant differences among these three groups, respectively.

**Table 2:** Correlations of metabolic parameters and clinicopathological characteristics with HER2 expression

	Total tumor numbers (*n* = 271)	HER2-zero (*n* = 62)	HER2-low (*n* = 120)	HER2-positive (*n* = 89)	*P* value
**Age**,** years (± SD)**		55 ± 12	55 ± 13	52 ± 12	0.17^a^
**BMI**		24.8 (22.7–27.7)	23.9 (22.1–26.1)	23.5 (21.0–26.5)	0.06^b^
**T stage**					0.39^c^
1/2	235 (86.7)	57 (91.9)	102 (85.0)	76 (85.4)	
3/4	36 (13.3)	5 (8.1)	18 (15.0)	13 (14.6)	
**Lymphatic metastasis**					0.16^c^
Absent	87 (32.1)	26 (41.9)	34 (28.3)	27 (30.3)	
Present	184 (67.9)	36 (58.1)	86 (71.7)	62 (69.7)	
**Distant metastasis**					0.02*^c^
Absent	244 (90.0)	61 (98.4)	108 (90.0)	75 (84.3)	
Present	27 (10.0)	1 (1.6)	12 (10.0)	14 (15.7)	
**Histologic grade**					0.04*^b^
Grade I	15 (5.6)	5 (8.1)	10 (8.3)	0 (0.0)	
Grade II	121 (44.6)	31 (50.0)	53 (44.2)	37 (41.6)	
Grade III	135 (49.8)	26 (41.9)	57 (47.5)	52 (58.4)	
**Tumor histology**					0.96^c^
Ductal	223 (82.3)	51 (82.3)	98 (81.7)	74 (83.1)	
Non-ductal	48 (17.7)	11 (17.7)	22 (18.3)	15 (16.9)	
**ER**					0.002*^c^
Negative	74 (27.3)	23 (37.1)	20 (16.7)	31 (34.8)	
Positive	197 (72.7)	39 (62.9)	100 (83.3)	58 (65.2)	
**PR**					0.002*^c^
Negative	116 (42.8)	28 (45.2)	38 (31.7)	50 (56.2)	
Positive	155 (57.2)	34 (54.8)	82 (68.3)	39 (43.8)	
**Ki-67**					0.01*^c^
< 30%	84 (31.0)	19 (30.6)	47 (39.2)	18 (20.2)	
≥ 30%	187 (69.0)	43 (69.4)	73 (60.8)	71 (79.8)	
**SUVmax**		4.81 (3.23–9.57)	6.07 (3.90–9.15)	7.15 (4.68–10.75)	0.049*^b^
**MTV**		7.17 (4.10–15.23)	6.59 (3.50–11.82)	6.21 (3.36–13.50)	0.55^b^
**TLG**		27.26 (10.08–54.56)	21.93 (9.94–48.66)	27.27 (12.54–60.68)	0.50^b^
**TLR**		1.90 (1.34–3.78)	2.46 (1.50–3.68)	2.96 (1.84–4.05)	0.03*^b^

Qualitative data are numbers followed by percentages in parentheses; continuous data are median followed by interquartile range (IQR) in parentheses, except for age. Hormone receptor (HR) has the same distribution as ER

Abbreviations: *HER2* human epidermal growth factor receptor 2, *BMI* body mass index, *ER* estrogen receptor, *PR* progesterone receptor, *SUVmax* maximum standardized uptake value, *MTV* metabolic tumor volume, *TLG* total lesion glycolysis, *TLR* tumor-to-liver SUV ratio

*P value < 0.05. ^a^analysis of variance (ANOVA); ^b^Kruskal-Wallis test; ^c^Chi-Squared test

**Fig. 2:**
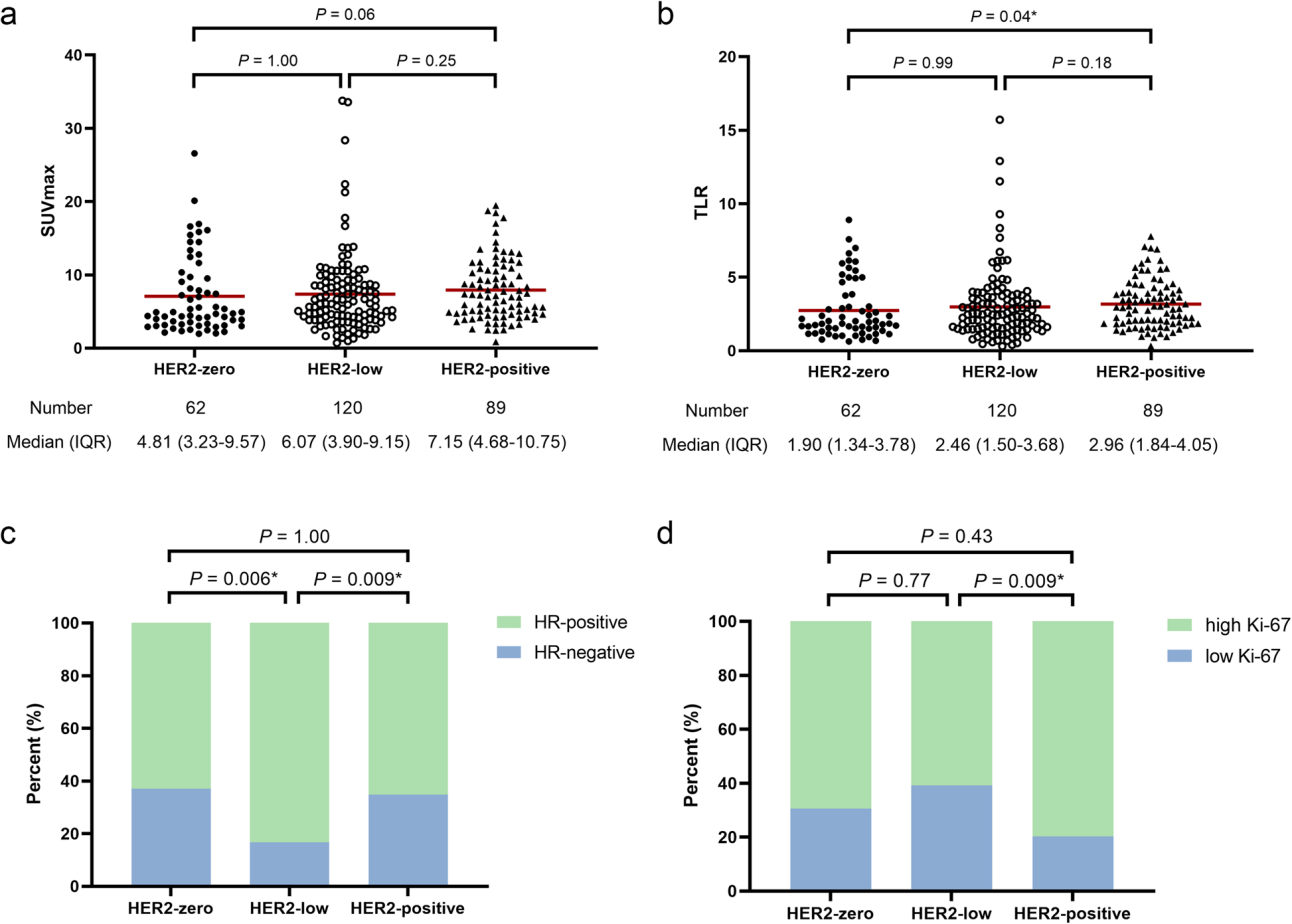
Multiple comparisons of maximum standardized uptake value (SUVmax) (**a**), tumor-to-liver SUV ratio (TLR) (**b**), hormone receptor (HR) (**c**), and Ki-67 (**d**) in different HER2 expression statuses. Ki-67 ≥ 30% was considered the threshold for the classification of high and low Ki-67. All the *P* values were corrected by Bonferroni correction. * Significant after Bonferroni correction

In multiple comparisons, TLR of HER2-positive group was significantly higher than that of HER2-zero group (*P* = 0.04), and SUVmax had the same tendency but was not significant after Bonferroni correction (*P* = 0.06) (Fig. 2a and b). Trends in metabolic parameters with HER2 expression (HER2-zero, -low and -positive) were analyzed by the Jonckheere-Terpstra test, which showed that *P* values for the four metabolic parameters were 0.01 (SUVmax), 0.43 (MTV), 0.43 (TLG), and 0.009 (TLR), respectively.

Additionally, the HR-positive rate of HER2-low group (83.3%) was significantly higher than both of HER2-zero group (62.9%, *P* = 0.006) and HER2-positive group (65.2%, *P* = 0.009), respectively (Fig. 2c). And the high Ki-67 rate of HER2-positive group (79.8%) was significantly higher than that of HER2-low group (60.8%, *P* = 0.009) (Fig. 2d).

### Subgroup analysis of metabolic characteristics between HER2-low and -zero under different HR statuses

As shown in Table 3, under the HR-positive status, both SUVmax and TLR exhibited significant differences between HER2-low and -zero (*P* = 0.045 and 0.03, respectively), indicating that the HR-positive HER2-low subgroup corresponded to relatively higher glucose metabolism compared to HR-positive HER2-zero subgroup. But, in the HR-negative subgroup (TNBC), no metabolic parameters indicated significant differences between HER2-low and -zero.

**Table 3:** Subgroup analysis of metabolic characteristics between HER2-low and HER2-zero under different HR statuses

	HR-positive (*n* = 139)			HR-negative (Triple-negative) (*n* = 43)		
	HER2-zero (*n* = 39)	HER2-low (*n* = 100)	*P* value	HER2-zero (*n* = 23)	HER2-low (*n* = 20)	*P* value
**SUVmax**	4.27 (3.07–5.62)	5.64 (3.71–8.79)	0.045*	7.87 (4.34–14.51)	7.36 (5.98–10.42)	0.85
**MTV**	6.02 (4.10–13.89)	7.14 (3.66–12.27)	0.89	9.09 (4.10–17.66)	4.22 (2.35–8.30)	0.06
**TLG**	18.60 (7.88–34.67)	21.41 (10.28–48.66)	0.44	31.18 (14.22–102.95)	23.42 (8.75–66.53)	0.18
**TLR**	1.66 (1.15–2.37)	2.32 (1.46–3.68)	0.03*	2.88 (1.96–5.45)	3.21 (2.26–4.20)	0.90

*P value < 0.05

Data are median followed by interquartile range (IQR) in parentheses. All using Mann-Whitney U test

Abbreviations: *HER2* human epidermal growth factor receptor 2, *HR* hormone receptor, *SUVmax* maximum standardized uptake value, *MTV* metabolic tumor volume, *TLG* total lesion glycolysis, *TLR* tumor-to-liver SUV ratio

### Metabolic characteristics of detailed HER2 expression

**Fig. 3:**
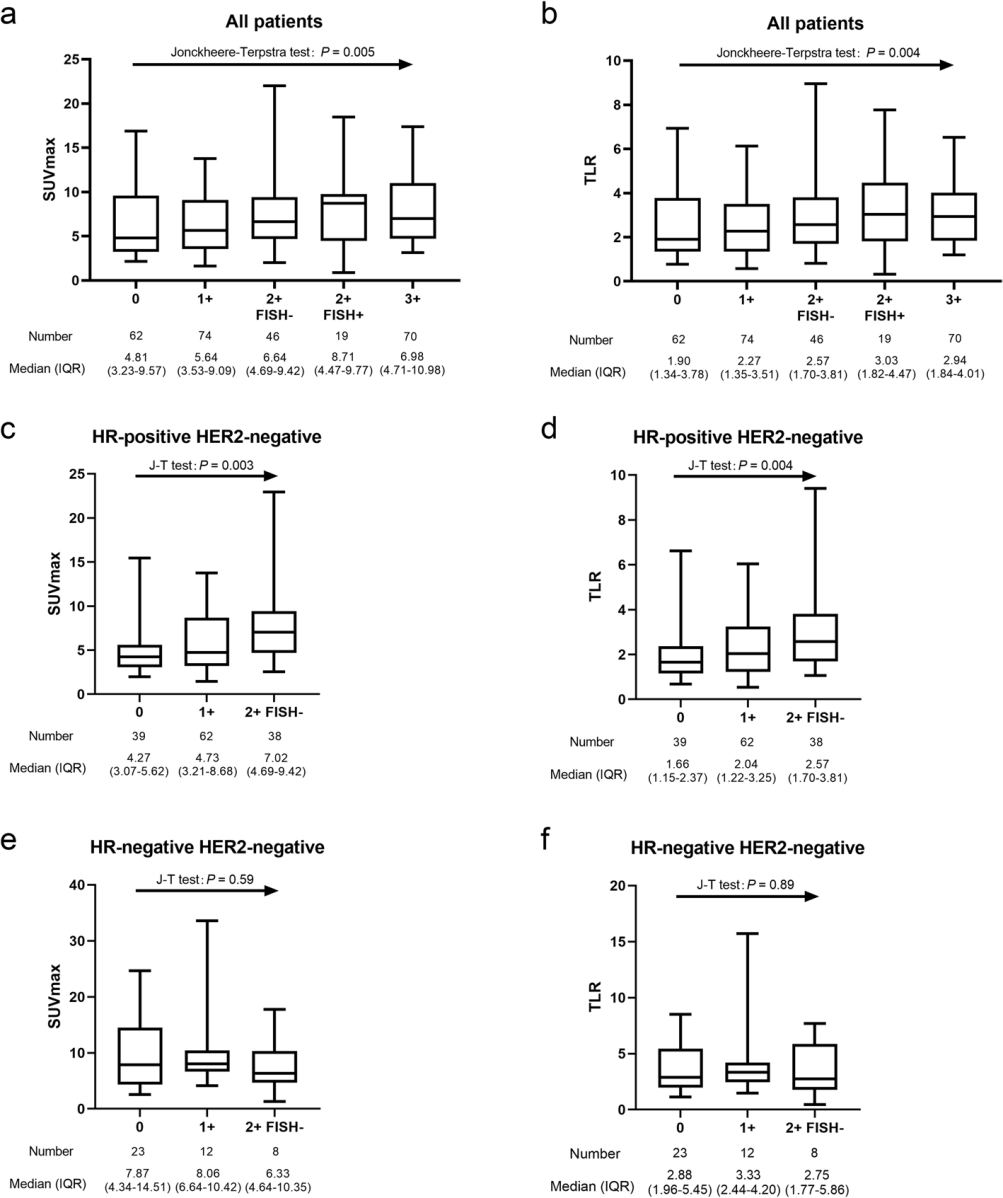
Analysis of maximum standardized uptake value (SUVmax) and tumor-to-liver SUV ratio (TLR) under detailed HER2 expression statuses in different groups. (**a** and **b**) all patients, (**c** and **d**) hormone receptor (HR)-positive HER2-negative group, (**e** and **f**) HR-negative HER2-negative group. Jonckheere-Terpstra test (J-T test) was used for trend testing

**Fig. 4:**
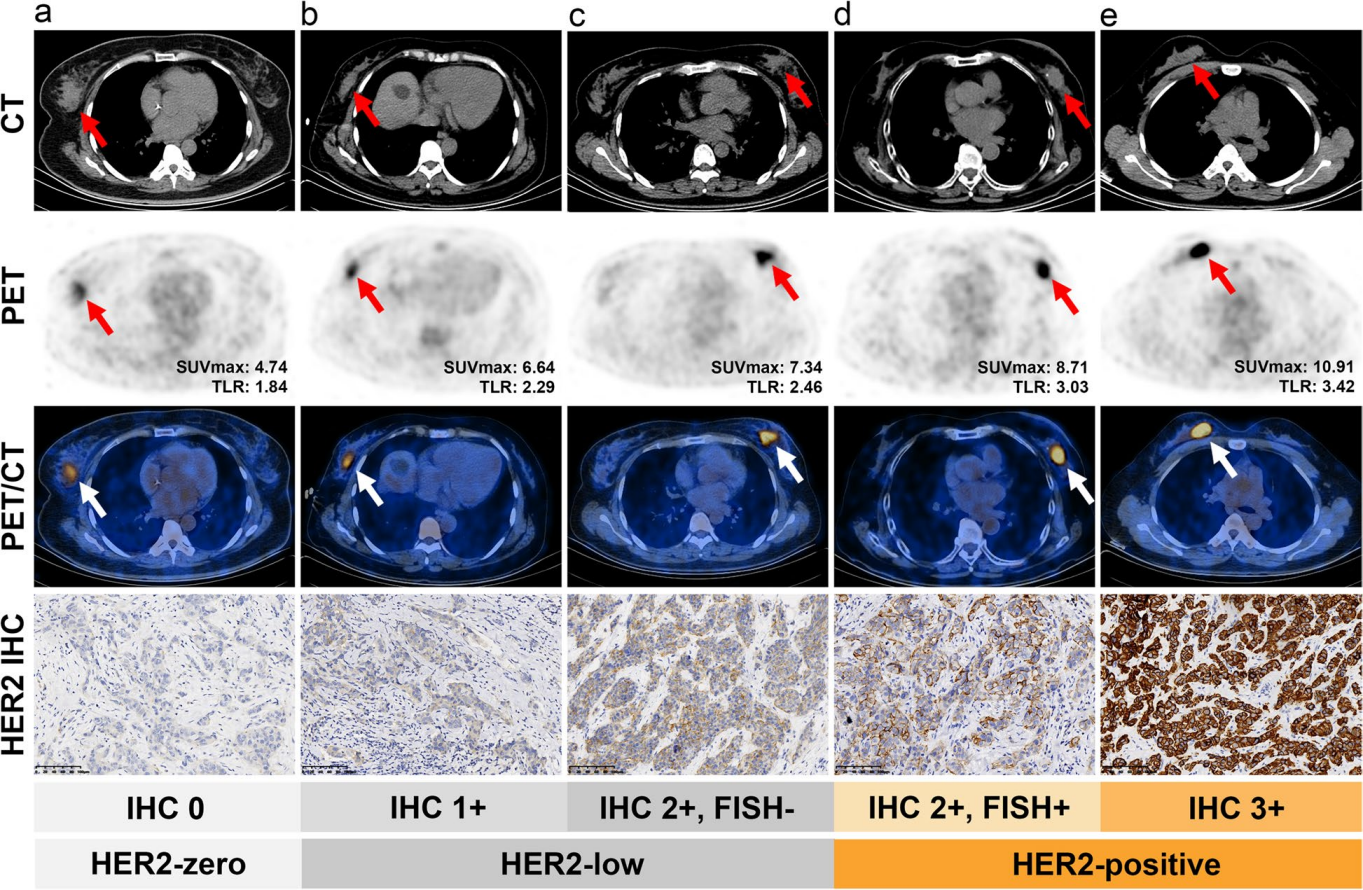
The typical images of glucose metabolism in primary lesions and their human epidermal growth factor receptor 2 (HER2) expression by immunohistochemistry (IHC). **a** A primary tumor with HER2-zero (IHC 0). **b** A primary tumor with HER2-low (IHC 1+). **c** A primary tumor with HER2-low (IHC 2 + with fluorescence in situ hybridization (FISH) (-)). **d** A primary tumor with HER2-positive (IHC 2 + with FISH (+)). **e** A primary tumor with HER2-positive (IHC 3+). The arrows indicated primary tumors. The scale of pathology images is 100 μm

We further explored the correlation of metabolic parameters (SUVmax and TLR) with different HER2 expression statuses. In all patients and in the HR-positive HER2-negative subgroup, SUVmax and TLR tended to increase with increasing HER2 expression (All the *P* values were less than 0.01 in Jonckheere-Terpstra test, Fig. 3a and b). However, this tendency was not shown in the HR-negative HER2-negative subgroup (i.e. triple-negative breast cancer, TNBC) (All the *P* values were more than 0.05 in Jonckheere-Terpstra test, Fig. 3c). Representative cases were displayed in Fig. 4.

### Prognostic analysis

**Fig. 5:**
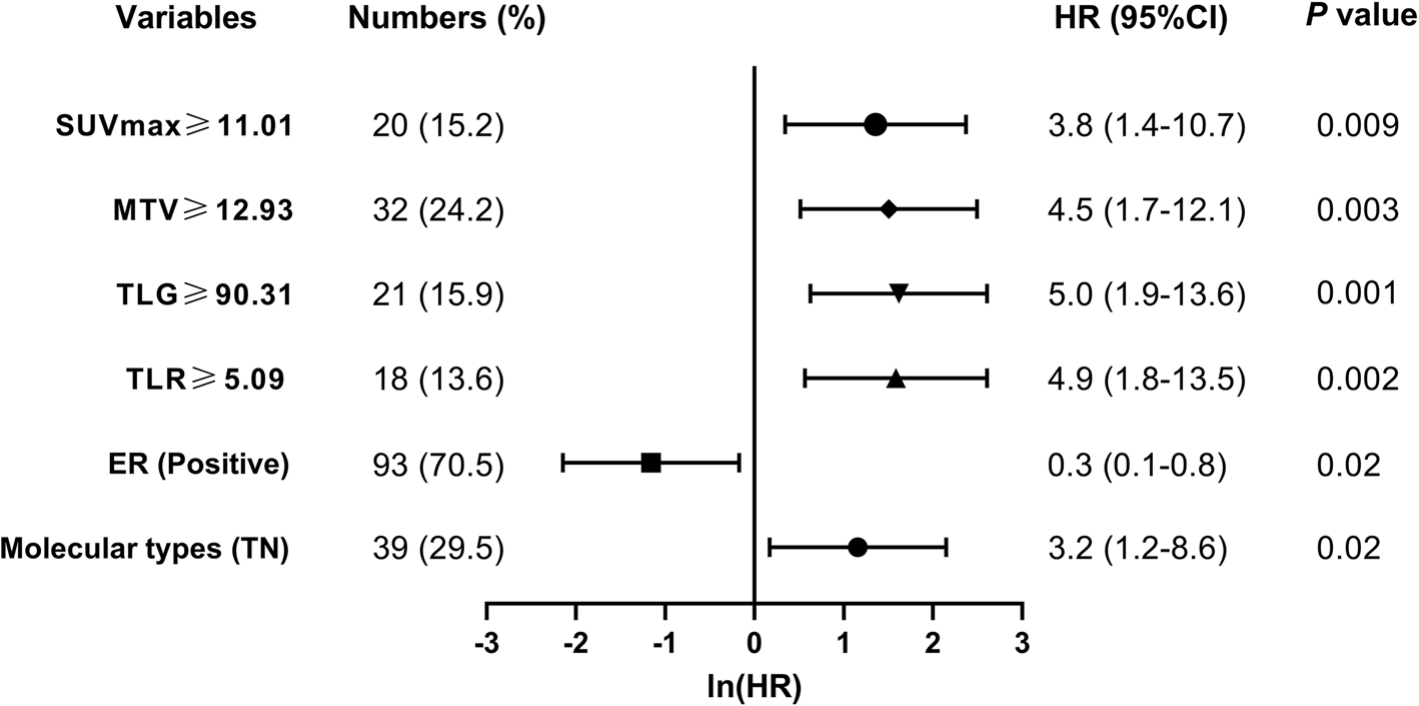
Forest plot of the prognostic predictors for disease-free survival (DFS) in HER2-negative patients using the univariate Cox hazards analysis. Receiver operating characteristic (ROC) curves were analyzed for the optimal cutoff value

General characteristics of patients with HER2-low and -zero breast cancer in prognostic analysis cohort were given in Table 4. In univariate Cox proportional hazards analysis, all of the four metabolic parameters were significant factors for predicting DFS (*P* < 0.01), and the remaining variables were not statistically significant, except for ER and molecular types (*P* = 0.02 for both) (Fig. 5).

**Table 4:** General characteristics of patients with HER2-negative breast cancer in prognostic analysis cohort

	Total numbers (*n* = 132)	Disease-free numbers (*n* = 116)	Disease-progressive numbers (*n* = 16)
**Age, years (± SD)**			
< 50	51 (38.6)	43 (37.1)	8 (50.0)
≥ 50	81 (61.4)	73 (62.9)	8 (50.0)
**BMI**‡			
< 25	85 (64.4)	76 (65.5)	9 (56.3)
≥ 25	47 (35.6)	40 (34.5)	7 (43.7)
**T stage**			
1/2	117 (88.6)	104 (89.7)	13 (81.3)
3/4	15 (11.4)	12 (10.3)	3 (18.7)
**Lymphatic metastasis**			
Absent	42 (31.8)	39 (33.6)	3 (18.8)
Present	90 (68.2)	77 (66.4)	13 (81.2)
**Histologic grade**			
Grade I/ II	67 (50.8)	60 (51.7)	7 (43.8)
Grade III	65 (49.2)	56 (48.3)	9 (56.2)
**Tumor histology**			
Ductal	111 (84.1)	97 (83.6)	14 (87.5)
Non-ductal	21 (15.9)	19 (16.4)	2 (12.5)
**ER**			
Negative	39 (29.5)	31 (26.7)	8 (50.0)
Positive	93 (70.5)	85 (73.3)	8 (50.0)
**PR**			
Negative	51 (38.6)	42 (36.2)	9 (56.2)
Positive	81 (61.4)	74 (63.8)	7 (43.8)
**HER2**			
Zero	50 (37.9)	42 (36.2)	8 (50.0)
Low	82 (62.1)	74 (63.8)	8 (50.0)
**Ki-67**			
< 30%	42 (31.8)	38 (32.8)	4 (25.0)
≥ 30%	90 (68.2)	78 (67.2)	12 (75.0)
**Molecular types**			
Luminal	93 (70.5)	85 (73.3)	8 (50.0)
Triple-negative	39 (29.5)	31 (26.7)	8 (50.0)
**Treatment**			
NAT	72 (54.5)	62 (53.4)	10 (62.5)
Non-NAT	60 (45.5)	54 (46.6)	6 (37.5)
**SUVmax**		5.69 (3.58–9.04)	7.97 (4.16–16.05)
**MTV**		7.01 (4.10–12.38)	13.82 (4.40–22.05)
**TLG**		24.62 (10.56–45.14)	35.87 (16.32–209.96)
**TLR**		2.36 (1.46–3.68)	2.74 (1.63–5.97)

Qualitative data are numbers followed by percentages in parentheses; continuous data are median followed by interquartile range (IQR) in parentheses, except for age. Hormone receptor (HR) has the same distribution as ER

Abbreviations: *HER2* human epidermal growth factor receptor 2, *BMI* body mass index, *ER* estrogen receptor, *PR* progesterone receptor, *NAT* neoadjuvant therapy, *SUVmax* maximum standardized uptake value, *MTV* metabolic tumor volume, *TLG* total lesion glycolysis, *TLR* tumor-to-liver SUV ratio

**Fig. 6:**
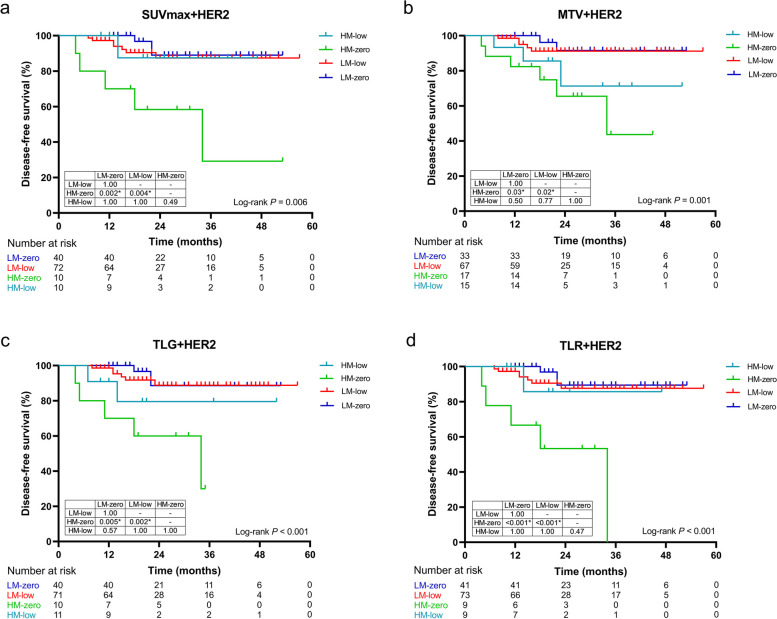
Kaplan-Meier survival curve analyses for disease-free survival (DFS) in HER2-negative patients. The cutoff values for distinguishing between high metabolism (HM) and low metabolism (LM) were 11.01 (**a**, maximum standardized uptake value (SUVmax)), 12.93 (**b**, metabolic tumor volume (MTV)), 90.31 (**c**, total lesion glycolysis (TLG)), and 5.09 (**d**, tumor-to-liver SUV ratio (TLR)), respectively. The bottom tables showed absolute numbers at risk. Tables below the survival curves showed the results of multiple comparisons, and *P* values have been corrected by Bonferroni correction. * Significant after Bonferroni correction

The prognostic cohort was further grouped according to metabolism and HER2 status for Kaplan-Meier analysis (Fig. 6). The results showed that there was no significant difference in DFS between HER2-low and -zero, regardless of high or low metabolism in the primary lesion. Furthermore, among HER2-zero patients, DFS was significantly shorter in those with high metabolism than in those with low metabolism, whereas there was no similar significant difference in HER2-low patients.

## Discussion

At present, anti-HER2 ADCs have opened up new therapeutic options for breast cancer patients with HER2-low, and there is increasing interest in the biological and clinical characteristics of this subgroup. However, the glucose metabolism characteristics and prognostic features of HER2-low based on pretreatment ^18^F-FDG PET/CT have not been well clarified. Our research attempted to comprehensively analyze whether baseline ^18^F-FDG PET/CT could reflect the metabolic features and disease outcomes in patients with HER2-low, especially when compared to patients with HER2-zero, in order to provide possible image-guided therapeutic strategies.

In our study, TLR showed significantly higher in the HER2-positive group than in the HER2-zero group. Additionally, HER2-low tumors were more frequent in HR-positive than HR-negative and were associated with lower Ki-67 statuses, which were consistent with prior reports [6, 28, 29]. In further exploratory analyses, we found that in both all patients and the HR-positive HER2-negative subgroup, SUVmax and TLR of the primary lesion tended to increase with the increase of HER2 expression (All the *P* values were less than 0.01), which suggested that the glucose metabolism of the different spectrum of HER2 expression might be a process of continuous evolution.

Specifically, SUVmax and TLR were significantly higher in HER2-low than in HER2-zero in the HR-positive subgroup (*P* = 0.045, *P* = 0.03, respectively), while there was no significant difference in HR-negative breast cancer (TNBC). Schettini et al. proposed that compared with HR-positive HER2-zero, HR-positive HER2-low tumors showed relatively lower expression of proliferation-related genes and three PAM50 (Prediction Analysis of Microarray 50) signatures, and higher expression of luminal-related genes and other two PAM50 signatures. But no individual gene or PAM50 signature was found differentially expressed between HER2-low and -zero in HR-negative breast cancer (TNBC) [30]. In addition, Shao et al. found that the pathologic complete response rates of HER2-low and -zero were significantly different in the HR-positive subgroup but not in HR-negative breast cancer (TNBC) [4]. Overall, the HR-positive HER2-low subtype may have a particularity in glucose metabolic profile, gene expression, and therapeutic response. Thus, it has the potential to be an independent subtype that deserves further exploration. As for HR-negative breast cancer (TNBC), we guessed that its highly invasive characteristics might mask metabolic differences between the HER2-low and -zero subgroups.

Most of the available studies aimed at comparing the differences in clinicopathological features and prognostic characteristics between HER2-low and -zero patients have yielded inconsistent results [4, 6, 31], with the majority of them showing no difference in prognosis between these two groups. However, there is a lack of research focusing on the prognostic role of metabolic parameters. Our study showed that the four included metabolic parameters were significant predictors of DFS in patients with HER2-negative (including HER2-low and -zero), but there was no significant difference in DFS between HER2-low and -zero, even under stratification of different metabolic levels.

In further stratified analysis, we found that the prognosis of HER2-zero patients with high metabolism was significantly worse than that of those with low metabolism, but no similar difference had been concluded in HER2-low patients. Recently, the concept of ultra-low HER2 expression (belong to the HER2-zero group) has received widespread attention from researchers [32], which is defined as having ≤ 10% of tumor cells with incomplete and weak staining despite an IHC score of zero [33]. In fact, the DESTINY-Breast06 trial has taken into account the potential benefits of ADC treatment for ultra-low HER2 patients and recently revealed the positive conclusion [34, 35].

On the other hand, more and more evidences suggest that the current definition of HER2-low by pathological examination does not seem to perfectly distinguish HER2 expression from non-HER2 expression, and its diagnostic accuracy in differentiating HER2-zero and HER2-low is also unsatisfactory [36, 37]. Our findings may provide complementary imaging indicators to help screen for patients with ultra-low HER2 who may benefit from ADC therapy. Of course, further investigations needed to determine whether ultra-low HER2 expression exists in HER2-zero patients with high metabolism, as well as to explore whether they may benefit from HER2-targeted ADC treatment.

Certainly, our study has several limitations that should be considered. First, our sample size was insufficient to thoroughly analyze the metabolic characteristics of various subgroups with different HER2 as well as HR status. The research on metastatic stage patients was also limited by a small sample size. At the same time, retrospective case collation may be prone to inadvertent bias. Second, because of the relatively good prognosis of non-metastatic breast cancer (5-year survival rate of approximately 90%) [38], we need to continue follow-up to further refine our prognostic findings, especially the subgroup analysis of different HR status. Third, radiomics based on ^18^F-FDG PET/CT imaging can provide more comprehensive imaging information and is expected to serve as an alternative method for detecting HER2-low expression. Last but not least, it is necessary to explore other more precise methods capable of evaluating HER2 in detail. HER2-targeted PET imaging is a possible option for determining the HER2 status [39].

## Conclusion

Our study revealed that the HR-positive HER2-low subgroup exhibited a particularity in glucose metabolic profile, which showed higher glucose metabolism compared with HR-positive HER2-zero. In addition, we found that HER2-zero breast cancer patients with elevated metabolic parameters had a poorer prognosis and warranted careful attention in clinical evaluations.

## Data Availability

No datasets were generated or analysed during the current study.

## Abbreviations

ADCs: Antibody drug conjugates
AUC: Area under the curve
CI: Confidence intervals
DFS: Disease-free survival
ER: Estrogen receptor
FDG: Fluorodeoxyglucose
FISH: Fluorescence in situ hybridization
HE: Hematoxylin and eosin staining
HER2: Human epidermal growth factor receptor 2
HR: Hormone receptor
HR: Hazard ratios
IHC: Immunohistochemistry
MRI: Magnetic resonance imaging
MTV: Metabolic tumor volume
PAM50: Prediction Analysis of Microarray 50
PET/CT: Positron emission tomography/computed tomography
PR: Progesterone receptor
ROC: Receiver operating characteristic
SUVmax: Maximum standardized uptake value
TLG: Total lesion glycolysis
TLR: Tumor-to-liver SUV ratio
